# Attitudes and barriers towards participation in an acupuncture trial among breast cancer patients: a survey study

**DOI:** 10.1186/1472-6882-14-7

**Published:** 2014-01-08

**Authors:** Jun J Mao, Tiffany Tan, Susan Q Li, Salimah H Meghani, Karen Glanz, Deborah Bruner

**Affiliations:** 1 Department of Family Medicine and Community Health, Perelman School of Medicine at the University of Pennsylvania, 3400 Spruce St, 2 Gates Building, Philadelphia, PA 19104, USA; 2 Center for Clinical Epidemiology and Biostatistics, Perelman School of Medicine at the University of Pennsylvania, Philadelphia, PA 19104, USA; 3 Abramson Cancer Center, Perelman School of Medicine at the University of Pennsylvania, Philadelphia, PA 19104, USA; 4 University of Pennsylvania School of Nursing, Philadelphia, PA 19104, USA; 5 Nell Hodgson Woodruff School of Nursing, Emory University, Atlanta, GA 30322, USA

**Keywords:** Acupuncture, Breast neoplasm, Clinical trial, Aromatase inhibitors/*adverse effects, Musculoskeletal, Joint pain, Attitudes, Barriers

## Abstract

**Background:**

As breast cancer patients increasingly use complementary and alternative medicine (CAM), clinical trials are needed to guide appropriate clinical use. We sought to identify socio-demographic, clinical and psychological factors related to willingness to participate (WTP) and to determine barriers to participation in an acupuncture clinical trial among breast cancer patients.

**Methods:**

We conducted a cross-sectional survey study among post-menopausal women with stage I-III breast cancer on aromatase inhibitors at an urban academic cancer center.

**Results:**

Of the 300 participants (92% response rate), 148 (49.8%) reported WTP in an acupuncture clinical trial. Higher education (p = 0.001), increased acupuncture expectancy (p < 0.001), and previous radiation therapy (p = 0.004) were significantly associated with WTP. Travel difficulty (p = 0.002), concern with experimentation (p = 0.013), and lack of interest in acupuncture (p < 0.001) were significant barriers to WTP. Barriers differed significantly by socio-demographic factors with white people more likely to endorse travel difficulty (p = 0.018) and non-white people more likely to report concern with experimentation (p = 0.024). Older patients and those with lower education were more likely to report concern with experimentation and lack of interest in acupuncture (p < 0.05).

**Conclusions:**

Although nearly half of the respondents reported WTP, significant barriers to participation exist and differ among subgroups. Research addressing these barriers is needed to ensure effective accrual and improve the representation of individuals from diverse backgrounds.

## Background

This year, an estimated 226,870 women will be diagnosed with breast cancer and most of them will join the 2.6 million breast cancer patients living in the United States [1]. Due to the extensive symptom distress experienced by this population and many women’s desire for natural approaches, many breast cancer patients use complementary and alternative medicine (CAM) [2-6]. Further, women may also turn to CAM therapies, as data suggests that these therapies may help patients ameliorate the side effects of treatment and the late effects of their disease [7-10]. Data suggests that the rates of CAM use have increased among women with breast cancer in recent years to as high as 84% [11-15].

In order to guide evidence-based use of CAM for breast cancer patients, clinical trials are needed to evaluate the safety, efficacy and effectiveness of these potential therapies, in particular, for symptom management. Effective recruitment to clinical trials is critical to the successful execution of trials; however about 38% of cancer clinical trials (CCT) fail to meet minimum accrual goals [16]. Further, 80% of trials are unable to achieve accrual goals within the anticipated recruitment period and remain open longer than planned, thus incurring additional costs and delaying the delivery of scientific findings to patients and clinicians [17,18].

Annually, very few adult cancer patients participate in CCTs, and racial/ethnic minorities are often underrepresented in CCTs [19]. Studies have shown that factors influencing participation in CCTs include participant demographics, insurance coverage, awareness of CCTs, potential side-effects of the CCT, trial setting, concern with the research process, complexity and stringency of the research protocol, and physician attitudes towards the trial [20-24]. Research examining accrual to CAM trials among breast cancer patients is extremely limited with only one study investigating factors affecting participation in a mind-body trial, which reported that 30% of its participants consented to participate in a CAM clinical trial [25]. Furthermore, few studies have examined participation, specifically, in symptom management trials. Instead, most aggregate total trial participation or focus on participation in therapeutic CCTs [19,21,26,27]. As Agrawal et al. found that fear of cancer growth is often the primary reason for clinical trial participation, it is clear that there may be differences in attitudes and barriers to trial participation between therapeutic and symptom management CCTs [28].

A better understanding of factors that affect participation in CAM CCTs is critically important for planning and executing successful investigations and ensuring adequate representation from historically underrepresented groups of individuals into these trials. Many of the CAM interventions such as acupuncture require weekly or, even more often, interventions over several weeks or months, thus the burden on potential research subjects from trial participation can be greater than conventional CCTs. In addition, most research to date has focused on studying enrollment towards CCTs for treatment of cancer, which may differ from CAM trials as CAM is often used for symptom management. Thus, we conducted this study to (1) identify the attitudes and barriers towards willingness to participate (WTP) in an acupuncture trial for joint pain among breast cancer patients; (2) determine the demographic, clinical, and psychological variables that may be predictive of WTP, and (3) elucidate the relationship between socio-demographic variables and perceived barriers to trial participation.

We chose acupuncture as the modality of focus because in a review of large U.S. comprehensive cancer center websites, Brauer et al. found that 60% of websites supported acupuncture as a means for symptom management in cancer; thus this therapy shows great promise for integration into conventional cancer care [29]. In addition, a population-based study found that cancer patients use acupuncture at a greater rate than non-cancer controls (10.2 vs. 6.2, p < 0.001) [2]. Aromatase-inhibitor (AI) related arthralgia was chosen as a symptom to focus on because we found that it affects close to 50% of women who take AI as adjuvant therapy for breast cancer [30].

## Methods

### Study design and patient population

We conducted a cross-sectional survey of breast cancer patients receiving care at the Rena Rowan Breast Center at the Abramson Cancer Center of the University of Pennsylvania (Philadelphia, PA) between April and October 2007 as part of a larger cohort study that is ongoing. Potential participants included all postmenopausal women with a history of histologically confirmed stage I to III, hormone-receptor-positive breast cancer who were currently taking a third-generation AIs (anastrozole, letrozole, or exemestane), had completed chemotherapy or radiotherapy at least one month prior to enrollment, and had the ability to understand and provide informed consent in English. Research assistants obtained permission from the treating oncologist, screened medical records and approached potential study subjects for enrollment at their regular follow-up appointments. After informed consent was obtained, each participant was given a self-administered survey. The study was approved by the Institutional Review Board of the University of Pennsylvania and the Scientific Review and Monitoring Committee of the Abramson Cancer Center.

### Survey instrument

The primary outcome measure was WTP in an acupuncture clinical trial among breast cancer patients with AI-related arthralgia. Participants were presented with a description of the study, “We are interested in performing an acupuncture study for joint pain among breast cancer patients receiving aromatase inhibitors. It will require up to 8–10 treatments over the course of 6–8 weeks. Each treatment lasts about 20 to 45 minutes. Acupuncture will be performed by a licensed acupuncturist. You will be compensated at the end of the study for your time and travel.” This was followed by a hypothetical question, “How likely are you to participate in an acupuncture trial, should you have daily bothersome joint pain?” Response options included “very much,” “somewhat,” “a little,” and “not at all.”

To assess the theoretical barriers to participation, participants were asked how much they agreed to statements regarding barriers to participation. These barriers were identified during focus group interviews, as well as from previous literature regarding CAM clinical trial recruitment [25,31-34]. Barriers surveyed included: travel difficulty, concern with experimentation (“I find it difficult to participate in an acupuncture study because I don’t want to be ‘experimented’ on”), presence of placebo group (“I find it difficult to participate in an acupuncture study because I don’t want to be in the placebo group”), demanding jobs, home responsibilities, and lack of interest in acupuncture. Participants chose from “strongly disagree,” “disagree,” “not sure,” “agree,” and “strongly agree.” We piloted the instrument before administration of the survey.

We also used the Acupuncture Expectancy Scale (AES), a validated instrument to measure outcome expectancy related to acupuncture, as belief in acupuncture may affect WTP in an acupuncture trial [35,36]. AES was previously validated in breast cancer patients with scores ranging from 4 to 20 with higher score indicating greater outcome expectancy. The internal consistency of the scale (Cronbach’s alpha coefficient) was 0.95 [36]. Other covariates measured included socio-demographic variables (i.e. age, race/ethnicity, education level, and employment status), and clinical and psychological factors (i.e. prior CAM use and severity of joint pain in the past 7 days), which were collected through self report. Clinical and treatment characteristics were also assessed by medical record abstraction (i.e. stage of breast cancer, previous surgery, previous chemotherapy, and previous radiotherapy). Traveling distance was calculated using Google Maps between home residence and hospital location.

### Statistical analysis

All data was entered by research assistants with verification by a separate data manager. Less than 5% of the data was missing in all of the key variables described in the paper. Missing data was treated as missing. Data analysis was performed using STATA 12.0 for Windows (STATA Corporation, College Station, TX). For WTP, we coded responses of “somewhat,” and “very much” to the question, “How likely are you to participate in an acupuncture trial, should you have daily bothersome joint pain?” Participants who selected “not at all” and “a little” were coded as not WTP. We chose to dichotomize this outcome to ease the interpretation of the data because the actual clinical trial participation is a dichotomous decision “yes” and “no.” This dichotomous variable was used as the main outcome in both bivariate and multivariate analyses. For the barriers to participation, we coded participants who “agree” and “strongly agree” to the barriers as endorsing the barriers. We initially computed descriptive statistics for all items. We then performed chi-square analyses to determine the relationship between WTP, relevant covariates (e.g. age, education, and race/ethnicity) and specific barriers. We also performed chi-square analyses to evaluate the relationship between key socio-demographic factors (i.e. age, race/ethnicity, and education status) and specific barriers. We then built multivariate logistic regression models in three steps with WTP as the dependent variable. Variables that were associated with the WTP at the significance level less than 0.10 in bivariate analyses were included. In Model 1, we sought to identify the demographic, clinical, and psychological factors related to WTP. In Model 2, we aimed to identify the barriers related to WTP. In Model 3, we incorporated factors that were significant in both models 1 and 2. Further, since joint pain may impact WTP, we repeated Model 3 analyses restricting only to those with current joint symptoms. All analyses were two-sided with p-value less than 0.05 indicating significance.

## Results

### Patient characteristics

As previously reported, 300 (92% of eligible patients) participated in this study [30]. Among those who declined participation (N = 25), common reasons were lack of time, not feeling well, and did not want to participate in research. Among the study participants, the mean age was 61.5 (SD = 9.9). The majority of the participants were non-Hispanic white people (84.3%) and a substantial proportion of non-Hispanic black (12.7%). Educational status varied among the participants: 40.7% had a high school level education or less, 25.6% had college-level education, and 33.7% had graduate or professional school level education. Other characteristics of the study participants can be seen in Table 1.

**Table 1:** Demographic and clinical characteristics of participants (N = 300)

	**Total respondents**	**Willing to participate**	**P-value**
	**N**	**%**	
**Total**	**297**	49.8	
**Age**			
≤55	71	60.6	***0.037***
56-65	130	50.8	
>65	96	40.6	
**Race/Ethnicity**			
White	250	51.2	0.28
Non-White	47	42.6	
**Educational level**			
High school or less	121	35.5	***<0.001***
College	76	69.7	
Graduate/professional school	99	51.5	
**Employment**			
Full-time	112	50.9	0.54
Part-time	40	42.5	
Not currently	141	52.5	
**Stage**			0.70
I	99	45.4	
II	141	49.6	
III	32	53.1	
**Chemotherapy**			0.17
No	118	44.9	
Yes	179	53.1	
**Radiation therapy**			***0.017***
No	106	40.6	
Yes	191	55.0	
**Type of AI**			**0.056**
Exemestane	57	43.9	
Anastrazole	171	46.8	
Letrozole	69	62.3	
**Months on AI**	23.5	21.7	0.11
**Prior acupuncture use**			***0.004***
No	271	47.2	
Yes	26	76.9	
**CAM user**			***<0.001***
No	114	36.8	
Yes	183	57.9	
**Co-morbidity**			***0.037***
None	86	59.3	
Yes	211	46.0	
**Joint pain during past 7 days**			***0.038***
None	54	35.2	
Mild/moderate	189	51.8	
Severe or more	53	58.5	
	**Mean for WTP**	**Std Dev.**	
**Acupuncture expectancy scale**	11.5	4.07	***<0.001***

*Abbreviations*: AI, Aromatase Inhibitor; CAM, complementary and alternative medicine.

*P-value obtained by Chi-2 for categorical and T-test for continuous outcomes.

Not all cells add up to 300 due to missing data.

### Socio-demographic, clinical, and psychological factors related to WTP

Among 300 participants, 148 (49.8%) were willing to participate in an acupuncture clinical trial. In the bivariate analysis, younger age, higher education, presence of co-morbidities, severe joint pain in the past 7 days and greater acupuncture expectancy were significantly associated with greater WTP (see Table 1). While stage of cancer, previous chemotherapy, and type of AI were not significantly related, previous radiation therapy and CAM use were significantly associated with greater WTP.

### Barriers to participating in an acupuncture trial

**Figure 1:**
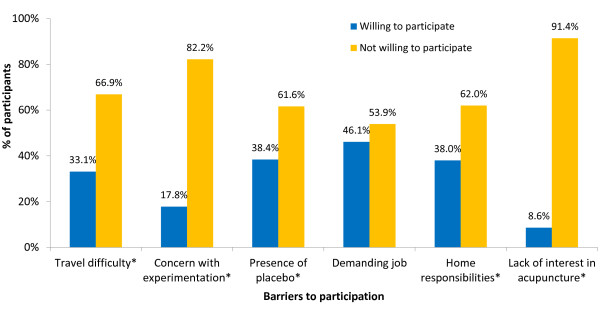
**Barriers to participation differ by willingness to participate.** *P < 0.001 by chi square analyses.

The most common barriers toward participation included presence of placebo group (45.9%), travel difficulty (45.6%), and home responsibilities (45%) followed by demanding job (35.6%), lack of interest in acupuncture (27.2%), and concern with experimentation (25.2%). In the bivariate analysis, all barriers but demanding job were significantly associated with WTP (p < 0.05) (see Figure 1).

Specific barriers to trial participation differed significantly across several key socio-demographic subgroups (see Table 2). In the bivariate analysis, older breast cancer patients were more likely to cite concern with experimentation (p < 0.001) and lack of interest in acupuncture (p = 0.008) as barriers toward trial participation, while younger women were more likely to cite demanding job (p < 0.001) and home responsibilities (p = 0.003) as barriers to participation. Similarly, employed women were more likely to consider demanding job (p < 0.001) and home responsibilities (p = 0.031) as barriers to participation. In addition, less educated women were more likely to consider concern with experimentation (p < 0.001), presence of placebo group (p = 0.005), and lack of interest in acupuncture (p = 0.001) as barriers to participation. Non-white women were more likely to consider concern with experimentation (p = 0.024) a barrier to participation. However, white women were more likely to cite travel difficulty (p = 0.018) as a barrier to participation. Among the survey participants, fewer white women lived within 10 miles of the hospital (17.8%) compared with non-white women (68.1%), (p < 0.001).

**Table 2:** Socio-demographic factors and barriers to participation*

	**Travel difficulty**			**Concern with experimentation**			**Don’t want placebo**			**Demanding job**			**Home responsibilities**			**No interest in acupuncture**		
	**N**	**%**	**p-value**	**N**	**%**	**p-value**	**N**	**%**	**p-value**	**N**	**%**	**p-value**	**N**	**%**	**p-value**	**N**	**%**	**p- value**
**Age**			0.90			***<0.001***			***0.063***			***<0.001***			***0.003***			***0.008***
≤55	34	47.9		13	18.3		29	40.9		42	59.2		44	62.0		14	19.4	
56-65	58	45.0		23	18.1		53	41.4		46	36.5		54	42.5		30	23.1	
>65	42	44.7		37	40.2		52	55.9		15	16.3		33	35.5		37	38.5	
**Race**			***0.018***			***0.024***			0.27			0.42			0.96			0.43
White	120	48.6		55	22.6		109	44.5		89	36.6		110	45.1		66	26.3	
Non-White	14	29.8		18	38.3		25	53.2		14	30.4		21	44.7		15	31.9	
**Education**			0.54			***<0.001***			***0.005***			0.18			0.40			***0.001***
High school**	59	48.8		49	40.8		68	56.2		35	29.7		57	47.5		47	38.5	
College	30	40.5		9	12.3		24	32.9		28	38.4		28	38.4		15	19.7	
Graduate	45	45.9		15	15.6		42	43.3		40	41.2		46	47.4		19	19.2	
**Employment**			0.24			***0.037***			**0.100**			***<0.001***			***0.031***			0.17
Full-time	53	48.2		21	19.1		45	40.9		79	71.2		59	53.2		27	23.9	
Part-time	21	53.9		6	15.8		14	35.9		13	33.3		20	51.3		15	38.5	
Not currently	57	40.4		43	31.2		72	51.8		10	7.4		51	37.2		35	24.7	

*Bivariate chi-square analysis.

**High school or less.

### Multivariate analyses: factors related to WTP

In multivariate analyses adjusting for socio-demographic and clinical factors and self-reported barriers (Model 3 in Table 3), we found that college level education (adjusted odds ratio [AOR], 4.24, 95% confidence interval [CI], 1.77-10.17, p = 0.001), previous radiation therapy (AOR, 2.72, 95% CI, 1.39-5.32, p = 0.004), and a higher score on the Acupuncture Expectancy Scale (AOR, 1.17, 95% CI, 1.08-1.26, p < 0.001) were significantly associated with increased likelihood to report WTP, while endorsement of travel difficulty (AOR, 0.36, 95% CI, 0.19-0.68, p = 0.002), lack of interest in acupuncture (AOR, 0.12, 95% CI, 0.04-0.35, p < 0.001) and concern with experimentation (AOR, 0.31, 95% CI, 0.13-0.78, p = 0.013) were barriers independently associated with decreased likelihood of participation. In a sub-analyses restricting to only those patients with current joint symptoms, we found very similar results. College education (AOR, 4.22, p = 0.003), previous radiation therapy (AOR, 2.39, p = 0.019), and higher expectancy (AOR, 1.16, p = 0.001) were significantly associated with greater WTP in acupuncture studies, while travel difficulty (AOR, 0.35, p = 0.003), concerns about experimentation (AOR, 0.34, p = 0.035), and lack of interest in acupuncture (AOR, 0.12, p = 0.001) were associated with decreased WTP in acupuncture.

**Table 3:** Factors related to willingness to participate: multivariate analysis

			Model 1*		**Model 2**^**+**^		**Model 3**^**++**^	
	**Bivariate analysis**		**Multivariate analysis**		**Multivariate analysis**		**Multivariate analysis**	
			**Pseudo R**^**2**^ **= 0.20**		**Pseudo R**^**2**^ **= 0.25**		**Pseudo R**^**2**^ **= 0.36**	
	**O.R. (95% C.I.)**	***p*-value**	**A.O.R. (95% C.I.)**	***p*-value**	**A.O.R. (95% C.I.)**	***p*-value**	**A.O.R. (95% C.I.)**	***p*-value**
**Age**								
≤55	1		1					
56-65	0.67 (0.37-1.21)	0.18	0.77 (0.38-1.58)	0.48				
>65	0.44 (0.24-0.83)	***0.011***	0.85 (0.40-1.83)	0.68				
**Education**								
High school or less	1		1				1	
College	4.18 (2.26-7.73)	< ***0.001***	4.07 (1.97-8.42)	***<0.001***			4.24 (1.77-10.17)	***0.001***
Graduate	1.93 (1.12-3.31)	***0.018***	1.86 (0.97-3.56)	0.062			1.27 (0.62-2.61)	0.51
**Radiation therapy**								
No	1		1				1	
Yes	1.79 (1.10-2.89)	***0.017***	2.09 (1.16-3.76)	***0.014***			2.72 (1.39-5.32)	***0.004***
**CAM use**								
No	1		1					
Yes	2.36 (1.46-3.82)	***<0.001***	1.26 (0.71-2.24)	0.43				
**Joint pain during past 7 days**								
None	1		1				1	
Mild/moderate	1.98 (1.06-3.71)	***0.032***	2.06 (0.96-4.44)	0.064			1.48 (0.61-3.57)	0.38
Severe or more	2.60 (1.19-5.67)	***0.017***	3.49 (1.34-9.04)	***0.01***			2.77 (0.89-8.59)	0.078
**Co-morbidity**								
No	1		1					
Yes	0.58 (0.35-0.97)	***0.038***	0.65 (0.34-1.23)	0.19				
**Acupuncture expectancy scale**	1.19 (1.12-1.26)	***<0.001***	1.20 (1.12-1.28)	***<0.001***			1.17 (1.08-1.26)	***<0.001***
**Travel difficulty**								
Disagree	1				1		1	
Agree	0.27 (0.16-0.44)	***<0.001***			0.41 (0.23-0.75)	***0.003***	0.36 (0.19-0.68)	***0.002***
**Concern with experimentation**								
Disagree	1				1		1	
Agree	0.13 (0.069-0.26)	***<0.001***			0.34 (0.14-0.81)	***0.015***	0.31 (0.13-0.78)	***0.013***
**Don't want placebo**								
Disagree	1				1			
Agree	0.40 (0.24-0.64)	***<0.001***			0.96 (0.50-1.83)	0.90		
**Demanding job**								
Disagree	1				1			
Agree	0.73 (0.45-1.18)	0.196			1.10 (0.58-2.09)	0.77		
**Home responsibilities**								
Disagree	1				1			
Agree	0.39 (0.24-0.62)	***<0.001***			0.77 (0.41-1.42)	0.40		
**Not interested in acupuncture**								
Disagree	1				1			
Agree	0.051(0.022-0.12)	***<0.001***			0.098 (0.040-0.24)	***<0.001***	0.12 (0.043-0.35)	***<0.001***

*Model 1: Multivariate analysis on sociodemographic and clinical factors related to willingness to participate.

^+^Model 2: Multivariate analysis on barriers related to willingness to participate.

^++^Model 3: Multivariate analysis on factors significant in Model 2 and 3 related to willingness to participate.

## Discussion

In this study, approximately one in two breast cancer patients were willing to participate in an acupuncture clinical trial for arthralgia. This figure is greater than WTP to general CCTs, which has been reported to range from 3% to 33% [19,26,37]. The most common barriers to participation in an acupuncture trial were travel difficulty, demanding jobs and home responsibilities, and concern with experimentation. Importantly, specific barriers differed by socio-demographic groups. Adjusting for relevant covariates, college education, previous radiation therapy, and higher expectancy of acupuncture outcomes were significantly associated with greater WTP. In addition, we identified barriers that were independently associated with decreased participation including travel difficulty, concern with experimentation, and lack of interest in acupuncture. These findings have implications for designing and executing acupuncture symptom management clinical trials to ensure timely and adequate accrual with sufficient representation of trial participants from diverse groups.

We found that those patients with a college education were more likely to report greater WTP in an acupuncture trial than those with high school or less education. Prior studies have indicated that higher education was related to both greater CAM use and more WTP in conventional cancer therapy clinical trials [23,32,33]. Thus, our study results were consistent with these findings. We also found that those with lower education were more likely to cite barriers such as concern with experimentation, presence of placebo, and lack of interest in acupuncture. To some degree, these factors may reflect lack of literacy regarding clinical trials and/or acupuncture; however, in multivariate models adjusting for these barriers, education was still highly associated with minimal change in adjusted odds ratio. These suggest factors related to education but not accounted for by these barriers, such as social support and income, may explain some of the association and warrant further investigation.

Higher expectancy of acupuncture outcomes for relief of arthralgia symptoms was associated with greater WTP in an acupuncture clinical trial. Although previous studies have investigated the association between expectancy and treatment outcome, to our knowledge, no prior studies have examined the relationship between outcome expectancy and WTP [38]. While clinical trials are meant to inform care delivery for future patients, it is not surprising that individuals who are interested in participating in a clinical trial do so in part with the hope that their specific conditions will be improved by trial participation. Given that previous research also found that higher expectancy of acupuncture outcomes may impact response to acupuncture, future clinical trials should include validated expectancy measures to help understand how expectancy may explain the variability observed in different studies [35,38-41].

Previous research has identified many barriers that present significant obstacles to accrual in conventional CCTs [21,23,31,34]. Our study not only identified barriers but also examined the how these barriers differed by specific socio-demographic factors, thereby identifying barriers to participation in symptom management trials. Our findings of ‘concern with experimentation’ and ‘travel difficulty’ as barriers to participation are similar to a recent meta-analysis by Mills et al. [21]. Non-white people, especially African Americans were significantly more likely than white people to cite concern with experimentation as a barrier, which indicates discomfort with the experimentation process [42,43]. This may be due to historical research abuses of blacks and the persistence of health inequities experienced by racial minorities [34,42,43]. Conversely, we found more white people reported travel difficulty as a barrier. This is likely due to the racial differences in geographic distance between a patient’s residence and the hospital among our study population. We found that more white people in our study lived farther away from the hospital, while more non-white people lived within a 10-mile radius. This is logical as the Hospital of the University of Pennsylvania is an inner city hospital surrounded by neighborhoods in which low income groups and minorities predominate. Our findings suggest that unique challenges to trial enrollment exist and different strategies are required to overcome barriers experienced by individuals of diverse socio-demographic groups.

There are a number of limitations that should be considered in the interpretation of our results. This study only examined hypothetical WTP in an acupuncture clinical trial. It is possible that these findings may not translate into actual clinical trial participation. Additionally, research using more sophisticated methods such as conjoint analyses may help better elucidate the complex decision making involved in trade-offs about different factors that drive patients’ willingness to enroll or not in a CAM trial. Our study also focused on studying only acupuncture for a specific symptom and should not be over-interpreted for other types of complementary and alternative medicine strategies. Further, our study was conducted in an academic medical center with majority of the participants being white which may limit its generalizability to community settings. Finally, African Americans were the predominant minority group included in this study thus limiting generalization of findings to other various vulnerable populations and racial minorities.

Despite the limitations, our study provided a comprehensive examination of factors associated with and barriers towards WTP in an acupuncture clinical trial. Furthermore, our study was unique in that it identified attitudes and barriers towards participation in a symptom management CCT. Previous studies mostly focused on studying participation in therapeutic cancer clinical trials. Thus our study provided novel information on participation in symptom management CCTs. As acupuncture and other mind-body CAM interventions (e.g. yoga, meditation) often require once or twice weekly interventions over multiple weeks, logistic issues such as traveling and competing responsibilities at home/work become substantially more important for these trials than the conventional CCT. Thus, having satellite sites to deliver the interventions, offering interventions at times that are convenient for participants, and offering baby-sitting may be some strategies to improve accrual and retention to these trials. Additionally, concerns about experimentation and lack of interest in acupuncture may be mitigated by appropriate education of potential research subjects about clinical trials and acupuncture, especially among the less educated or racial minorities, so that adequate representations from these groups can be achieved in CAM trials.

## Conclusions

We found that one in two breast cancer patients reported willingness to participate in an acupuncture trial for arthralgia. It is encouraging that a large proportion of patients are willing to participate in an acupuncture trial for symptom management which indicates acupuncture having clinical relevance in this population. Our findings of socio-demographic factors related to barriers will further allow researchers to strategically address the specific barriers faced by patients and ensure adequate representation in trials by individuals from historically underrepresented groups. With carefully designed and well-executed clinical trials, we can move towards an evidence-based integration of acupuncture for symptom management in cancer for individuals from diverse backgrounds.

## Abbreviations

AI: Aromatase inhibitor; AES: Acupuncture Expectancy Scale; CAM: Complementary and alternative medicine; CCT: Cancer clinical trial; WTP: Willingness to participate.

## Competing interests

The authors declare that they have no competing interests.

## Authors’ contributions

JJM conceived of the study and participated in its design and coordination and drafted the manuscript. TT analyzed and interpreted the data and helped draft the manuscript. SQL performed statistical analyses and drafted the manuscript. SHM, KG and DB participated in the design of the study, data interpretation, and helped revise the manuscript. All authors read and approved the final manuscript.

## Pre-publication history

The pre-publication history for this paper can be accessed here:

http://www.biomedcentral.com/1472-6882/14/7/prepub
